# *Brassica rapa* L. Polysaccharides Alleviate T2D via Modulation of Gut Microbiota and Metabolites

**DOI:** 10.3390/foods14244286

**Published:** 2025-12-12

**Authors:** Wei Li, Xinyao Han, Wei Wang, Qingping Du, Mingxun Ai, Shihao Huang, Tongle Sun, Hongji Zeng, Yuhang Li

**Affiliations:** College of Food Science and Pharmacy, Xinjiang Agricultural University, Urumqi 830052, China; liweili2025@163.com (W.L.);

**Keywords:** polysaccharide, type 2 diabetes, intestinal microorganism, amino acids, non-targeted metabolomics

## Abstract

The present research sought to investigate the impacts of *Brassica rapa* L. polysaccharide (Brp) on glycolipid metabolism in diabetic rats and the regulatory role of gut microbiota in metabolic balance. After 30-day Brp gavage, glycolipid metabolic parameters and amino acid levels were measured, and gut microbial communities were sequenced and analyzed. The results showed that Brp improved glycolipid metabolism, alleviated insulin resistance and hepatic oxidative stress, increased liver glycogen synthesis, and modulated gut microbiota composition. Specifically, Brp potentially enhanced short-chain fatty acid (SCFAs) production by enriching *Blautia* and *Roseburia* populations while reducing lipopolysaccharide (LPS)-producing bacteria to lower pathological risks. Notably, Brp may reduce the risk of T2D by increasing the concentration of lysophosphatidic acid (18:2(9Z,12Z)/0:0), indoleacrylic acid, cholic acid, and betaine, and decreasing high-risk metabolites such as 3,4-dihydroxybutyrate, xanthine, and carnitine, as well as regulating branched-chain amino acids and aromatic amino acids throughout the development of T2D.

## 1. Introduction

Diabetes mellitus constitutes a chronic metabolic disorder characterized by the body’s failure to produce sufficient insulin, inability to effectively utilize insulin, or a combination of both conditions [1]. Type 2 diabetes (T2D) represents a specific subtype of diabetes mellitus, characterized by the combined effect of “insulin resistance” and “β-cell dysfunction” [2]. T2D is initially dominated by insulin resistance, but in advanced stages, due to the “compensatory failure” of β cells, blood sugar becomes uncontrolled, which accounts for over 90% of diabetic cases [3]. T2D-related complications also amplify the risks of the disease, including vision loss, kidney failure, lower extremity amputation, cardiovascular disorders, and hepatic ailments [4]. While drug therapies, including metformin, DPP-4 inhibitors, SGLT-2 inhibitors, and GLP-1 agonists, can maintain fasting blood glucose levels within the normal range (70–99 mg/dL), these medications may trigger adverse effects like vitamin B12 deficiency, digestive system disturbances, liver toxicity, and lactic acidosis [5,6].

Recent research has revealed that natural polysaccharides possess notable anti-diabetic properties with few side effects or adverse reactions. Examples include *Magnolia officinalis* polysaccharides, *Panax notoginseng* leaf polysaccharides, edible bolete (*Phlebopus portentosus*) polysaccharides, Imperatae Rhizoma polysaccharides, *Spirulina platensis* polysaccharides, and *Polygonatum sibiricum* polysaccharides [7,8,9,10,11,12]. *Brassica rapa* L., a tuberous root plant, primarily grown in high-elevation regions of China (e.g., the Tianshan Mountains and Tibet), functions as both a staple vegetable and a traditional medicinal resource [13]. Its roots are abundant in glucosinolates and their isothiocyanate metabolites, with well-documented therapeutic attributes including detoxification, anti-cancer activity, and blood pressure regulation [14]; contemporary research has further focused on its other bioactive compounds like polysaccharides, which exhibit anti-fatigue, antioxidant, hypoglycemic, and immunomodulatory properties [15,16]. As a seasonal plant harvested exclusively in late autumn, traditional processing methods (e.g., pickling, vegetarian wrap production) are employed to extend its shelf life, yet surplus or lower-grade roots (e.g., misshapen, undersized, or slightly damaged) are often discarded due to strict market quality standards—these underutilized roots represent a cost-effective, sustainable raw material for extracting bioactive compounds or developing value-added products.

Intestinal bacteria influence host amino acid availability through two primary mechanisms: modulation of dietary substrate metabolism and direct production of absorbable amino acids [17]. Notably, certain bacteria-derived amino acids function as precursors in short-chain fatty acid synthesis, which has been linked to adiposity [18]. Given the correlation between concentrations of specific amino acids and metabolic perturbations in obesity and T2D rats [19,20], comprehensive understanding of amino acid metabolism is essential for elucidating global metabolic control mechanisms.

Given the limited studies elucidating the specific mechanism underlying *Brassica rapa* L. polysaccharide (Brp)’s hypoglycemic efficacy, this study assessed the therapeutic impact of Brp on T2D rats with insulin resistance, which was constructed by administering a high-fat diet (HFD) together with streptozotocin (STZ) injection. Brp’ hypoglycemic activity was evaluated by integrating blood glucose and lipid profile changes. Additionally, the underlying hypoglycemic mechanism of Brp was examined via 16S rRNA sequencing and metabolomics analyses.

## 2. Materials and Methods

### 2.1. Materials

Polysaccharide was obtained from *Brassica rapa* L., whose roots were purchased from the farmer’s market (Urumqi, Xinjiang, China). Brp, the polysaccharide fraction from *Brassica rapa* roots is primarily composed of fructose, rhamnose, arabinose, galactose, and galacturonic acid, with molar ratios of 0.81:4.30:3.61:1.69:89.59, and has a molecular weight distribution of 32.55 kDa. Brp was extracted via hot water extraction (60 °C, 1 h), followed by aqueous-ethanol fractionation (85% ethanol precipitation), deproteinization using the Sevag method, and purification with DEAE-52 cellulose and Sephadex G-100 columns. Notably, the current ultrasound-assisted hydroalcoholic extraction may face scalability limitations; thus, hydrodynamic cavitation could be evaluated for future large-scale Brp production [21]. The Brp used in this study was a new batch prepared using the identical method as the one described in Zhang et al. [22], ensuring consistency with previous work. And more detailed structural characterization of these turnip polysaccharides is reported in Zhang et al. [22]. Streptozotocin (STZ) was purchased from McLain, triglycerides (TG, S03027), total cholesterol (TC, S03042), high density lipoprotein cholesterol (HDL–C, S03025), low-density lipoprotein cholesterol (LDL-C, S03029), total superoxide dismutase (T-SOD, G4306), malondialdehyde (MDA, G4302) and glutathione peroxidase (GSH-Px, G4310) and assay kit including ELISA kits of serum insulin and hepatic glycogen were determined using assay kits from Wuhan Service Biotechnology Co., Ltd. (Wuhan, China). All other reagents were purchased from Shanghai Sangon Biotech Co., Ltd. (Shanghai, China) and Wuhan Service Biotechnology Co., Ltd. All utilized chemicals met analytical grade standards. Valine (Val, 72-18-4) and leucine (Leu, 61-90-5) were bought from Sigma-Aldrich (St. Louis, MO, USA); phenylalanine (Phe, 63-91-2), tyrosine (Tyr, 60-18-4), and tryptophan (Trp, 73-22-3) were purchased from Sinopharm Co., Ltd. (Beijing, China); isoleucine (Ile, 73-32-5) was purchased from Aladdin Reagent Co., Ltd. (Shanghai, China).

### 2.2. Experimental Animals and Design

Twelve 28-day-old male Sprague-Dawley (SD) rats, with an average weight of 180 g, were sourced from Xinjiang Medical University (Xinjiang, China). The rats were maintained in a controlled environment (25 °C, regulated humidity) with a 12:12 light-dark cycle for a 5-day acclimatization period. Following this, they were randomly allocated to different groups and designated as: NC (Normal group, n = 3) and model group (n = 9). The standard normal diet was administered to the NC group, whereas the model group received a high-fat diet (HFD) over a 4-week duration. After successful model establishment, the model rats were stratified by fasting blood glucose (FBG) levels and then randomly assigned to the model group without treatment (T2D, n = 3), the Metformin group (MET, n = 3), and the *Brassica rapa* L. polysaccharide group (Brs, n = 3) to balance the baseline FBG differences between groups.

The T2D model was constructed as per the protocol established by Liu et al. [23] with certain adjustments incorporated, using a combination of high-sugar and high-fat diet and STZ reagent for induction. Standard normal diet and high-fat diet were bought from Jiangsu Medicience Biomedical Co., Ltd. (Lianyungang, China), and the high-fat diet was composed of 54% rodent maintenance feed, 10% sucrose, 6% whole milk powder, 10% soy protein isolate, and 20% lard. After 28 days of high-sugar and high-fat induction, the model group received intraperitoneal STZ (35 mg/kg) injections using 0.1 M sodium citrate buffer (pH 4.4) as the solvent, while NC rats were provided with an equivalent volume of 0.1 M citrate buffer. FBG was measured on the 3rd and 7th days after the injection period, and modeling was considered successful if FBG ≥ 11.1 mmol/L. If modeling was unsuccessful, the above steps were repeated three days later. On the third and last day of the induction week, the fasting blood glucose (FBG) of each rat was monitored after fasting for 12 h. Rats with FBG greater than 11.1 mmol/L in both measurements were considered successfully established T2D models. Rats with fasting blood glucose (FBG) levels < 11.1 mmol/L in both measurements were excluded and replaced with reserve rats. Meanwhile, the model group was randomized into three cohorts as follows: the T2D group (administered with normal saline), Brs group (administered with Brp at 30 mg/kg via gavage at a volume of 10 mL/kg), and the MET group (administered with metformin at 300 mg/kg via gavage at a volume of 10 mL/kg). These doses were chosen based on [22,23], as these regimens have been shown to reliably induce T2D and demonstrate hypoglycemic effects in rodent models. This animal experimental procedure was rigorously performed following the operational guidelines of the Animals (Scientific Procedures) Act 1986, its associated regulations, and the EU Directive 2010/63 on the protection of animals used in scientific procedures. In addition, the experimental protocol gained clearance from the Institutional Animal Care and Use Committee (IACUC) of Xinjiang Agricultural University (Permit No. SCXK (Xin) 2023-0001) following formal review.

### 2.3. Collection of Samples

Following the interference test period, all rats were fasted for 12 h with free access to water to eliminate the interference of diet on detection indicators. After anesthesia with 3% sodium pentobarbital via intraperitoneal injection (dose: 50 mg/kg body weight) and confirmation of successful anesthesia, the abdominal cavity was quickly opened to expose the abdominal aorta, and whole blood was collected using a disposable sterile syringe. Immediately after blood collection, the rats were euthanized by cervical dislocation to achieve a humane endpoint. The collected blood samples were then immediately precooled in a 4 °C refrigerator for 30 min, followed by centrifugation at 3000 rpm for 15 min to separate serum. The serum was aliquoted into enzyme-free EP tubes, labeled, and stored long-term in a −80 °C ultra-low temperature freezer for subsequent biochemical index detection. The intact liver was excised, rinsed with pre-chilled normal saline to remove surface blood (to prevent tissue damage), and blotted dry with sterile filter paper. Each liver was bisected longitudinally along the median lobe: One part was quickly placed in 4% paraformaldehyde fixative for pathological section preparation; the other part was placed in a pre-chilled cryovial, immediately snap-frozen in liquid nitrogen, and then transferred to a −80 °C freezer for subsequent tests of physiological and biochemical indicators (e.g., enzyme activity, liver glycogen content). Each rat’s cecum was opened, and fresh fecal specimens were aseptically harvested from the cecal lumen using sterile forceps. The feces were collected individually (one fecal sample per rat, no cage-level pooling) from the cecal lumen of each rat using sterile forceps, quickly transferred to pre-chilled sterile cryovials, and immediately snap-frozen in liquid nitrogen. Finally, the individual fecal samples (3 samples per group, no pooling) were cryopreserved at −80 °C for subsequent analysis of gut microbiota and their metabolites.

### 2.4. Physiological and Insulin-Related Parameters

FBG (fasting blood glucose) and body weight were measured and recorded every 5 days. After an overnight fast, FBG was measured using a standard glucometer via tail vein puncture. On the last day of the intervention trial, an OGTT (oral glucose tolerance test) was performed every half an hour following an overnight fast [24]. Serum insulin (InS) levels were assayed with a commercial ELISA kit, while insulin resistance (HOMA-IR) and insulin sensitivity index (ISI) were computed using established formulas:

(1) HOMA−IR=InS×FBG22.5

(2) $$ISI=Ln(FBG\times {InS}^{-1})$$

### 2.5. Liver-Related Parameters

The hepatic levels of T-SOD, MDA, GSH-Px and glycogen were quantified following the methodology in the producer’s guideline. Specifically, T-SOD activity was measured via the xanthine oxidase method: the reaction system (240 μL total volume) contained tissue homogenate, xanthine substrate, and enzyme working solution; absorbance was recorded at 450 nm, and activity was expressed as U/mgprot. GSH-Px activity was determined using the dithionitrobenzoic acid (DTNB) method: the reaction system (0.5 mL total volume) included tissue homogenate, GSH substrate, and H_2_O_2_; absorbance was measured at 412 nm, and activity was calculated as U/mgprot.

### 2.6. Lipid Profile

Commercial kits were employed to assay rat serum levels of triglycerides (TG), total cholesterol (TC), LDL-cholesterol (LDL-C), and HDL-cholesterol (HDL-C) in strict adherence to manufacturer specifications.

### 2.7. Tissue Section

Liver tissues were harvested immediately after rat euthanasia and fixed in 10% neutral buffered formalin (NBF) at room temperature for 24 h (fixative-to-tissue volume ratio = 10:1) to preserve histological structural integrity. Fixed tissues were dehydrated through a graded ethanol series: 70% ethanol (2 h), 80% ethanol (2 h), 95% ethanol (2 h, twice), and 100% ethanol (1 h, twice). This was followed by clearing in xylene (1 h, twice) and infiltration with molten paraffin wax (58–60 °C, 2 h, twice). Tissues were embedded in paraffin blocks and sectioned into 4 μm-thick slices using a Leica RM2235 rotary microtome (Leica Biosystems, Wetzlar, Germany). Sections were deparaffinized in xylene (10 min, twice) and rehydrated through a reverse ethanol series before hematoxylin-eosin (H&E) staining. Stained sections were subjected to whole-slide scanning using a 3DHISTECH (Budapest, Hungary) PANNORAMIC DESK/MIDI/250/1000 whole-slide scanner. Scanned images were observed, annotated, and analyzed using 3DHISTECH CaseViewer v2.4 scanning and browsing software.

### 2.8. Gut Microbiota Analysis

The 16S *rRNA* amplicon sequencing was performed by Genesky Biotechnologies Inc., Shanghai, China. Briefly, total genomic DNA was extracted using the FastDNA SPIN Kit for Soil (MP Biomedicals, Santa Ana, CA, USA), according to the manufacturer’s instructions. The integrity of genomic DNA was detected through agarose gel electrophoresis, and the concentration and purity of genomic DNA were detected through the Nanodrop 2000 (Thermo Scientific, Waltham, MA, USA) and Qubit3.0 Spectrophotometer (Invitrogen, Carlsbad, CA, USA). The V3–V4 hypervariable regions of the 16S *rRNA* gene were amplified with the primers 341F (5-CCTAT2DGGNT2DCWGCAG-3) and 805R (5-GACTACHVGGGTATCTAATCC-3) and then sequenced using an Illumina NovaSeq 6000 sequencer (Illumina, San Diego, CA, USA). The V3–V4 hypervariable regions were subjected to PCR amplification with the 341F/805R primer set, followed by purification and paired-end sequencing (250 bp) on the Illumina NovaSeq 6000 platform. Raw sequencing data were processed using QIIME 2 [25]. The adaptor and primer sequences were trimmed using the cutadapt plugin. The DADA2 plugin was used for quality control and to identify amplicon sequence variants (ASVs) [26]. Taxonomic assignments of ASV representative sequences were performed with a confidence threshold of 0.7 by a pretrained Naive Bayes classifier, which was trained on the SILVA (version 138.2).

### 2.9. Untargeted Metabolomics Analysis

Metabolomic profiling utilized a Thermo Vanquish UHPLC system fitted with an ACQUITY UPLC^®^ HSS T3 column (2.1 × 100 mm, 1.8 μm; Waters, Milford, MA, USA). Isocratic separation was performed at a column temperature of 40 °C, with the mobile phase flowing at a rate of 0.3 mL per minute and each sample injection volume set to 2 μL [27]. For LC-ESI(+)-MS, the mobile phase comprised 0.1% formic acid in acetonitrile/aqueous formic acid; LC-ESI(−)-MS used acetonitrile/5 mM ammonium formate buffer [27].

Metabolite profiling employed an Orbitrap Exploris 120 mass spectrometer (manufactured by Thermo Fisher Scientific) equipped with an electrospray ionization (ESI) source. Key parameters: sheath/auxiliary gas flows = 40/10 arb, ion transfer capillary = 325 °C, spray voltage = +3.50 kV (ESI^+^)/−2.50 kV (ESI^−^). Full-scan MS data acquisition approach was implemented with a resolving power of 60,000 at a mass-to-charge ratio (*m*/*z*) of 200. Data-dependent MS/MS (ddMS^2^) was performed on the top 4 precursor ions with a resolution of 15,000 and dynamic exclusion enabled.

### 2.10. Amino Acids Metabolism

Stock solutions were prepared by accurately dissolving weighed standards in 0.1% (*v*/*v*) aqueous formic acid. Mixed standards were prepared by pipetting volumes of stock solutions and then diluted to desired concentrations with 10% formic acid–methanol–water (1:1, *v*/*v*) to obtain working standards. Fecal samples (2 mL centrifuge tubes) were mixed with 600 μL 10% formic acid-methanol-water (1:1, *v*/*v*), homogenized with two steel ball bearings (vortex 30 s, tissue grinder 55 Hz for 90 s), and centrifuged (12,000 RPM, 4 °C, 5 min). Supernatants were diluted 50-fold with 10% formic acid–methanol–water (1:1, *v*/*v*), vortexed (30 s), and 100 μL aliquots were mixed with 100 μL 20 ng/mL Trp-d3 internal standard, vortexed (30 s), filtered (0.22 μm), and transferred to vials for analysis.

Amino acid profiling was conducted using an AB Sciex ExionLC UHPLC system (ACQUITY UPLC^®^ BEH C18 column, 2.1 × 100 mm, 1.7 μm) with a gradient elution program: 0–6.5 min, 90% to 70% B; 6.5–7 min, 70% to 0% B; 7–14 min, 0% B; 14–14.5 min, 0% to 90% B; 14.5–17.5 min, 90% B; flow rates: 0–8.0 min (0.3 mL/min), 8.0–17.5 min (0.4 mL/min); mobile phases A (50% methanol-water + 0.1% formic acid) and B (10% methanol–water + 0.1% formic acid). Metabolite detection used an AB Sciex AB 6500 Plus mass spectrometer (ESI source) with parameters: ion source temperature 500 °C, voltage 5500 V, collision gas 6 psi, curtain gas 30 psi, and nebulizer/auxiliary gas 50 psi each (Sciex, Framingham, MA, USA). Multiple reaction monitoring (MRM) was employed for scanning.

### 2.11. Statistical Analysis

Statistical analysis was performed using SPSS 17.0 software (SPSS Inc., Chicago, IL, USA), where one-way analysis of variance (ANOVA) was employed to evaluate the data, followed by Tukey’s post hoc test for intergroup pairwise comparisons. All outcomes are expressed as mean ± standard deviation (SD), with statistical significance established when *p* ≤ 0.05.

## 3. Results

### 3.1. Impacts of Brp on T2D Physiological and Insulin-Related Parameters

**Figure 1:**
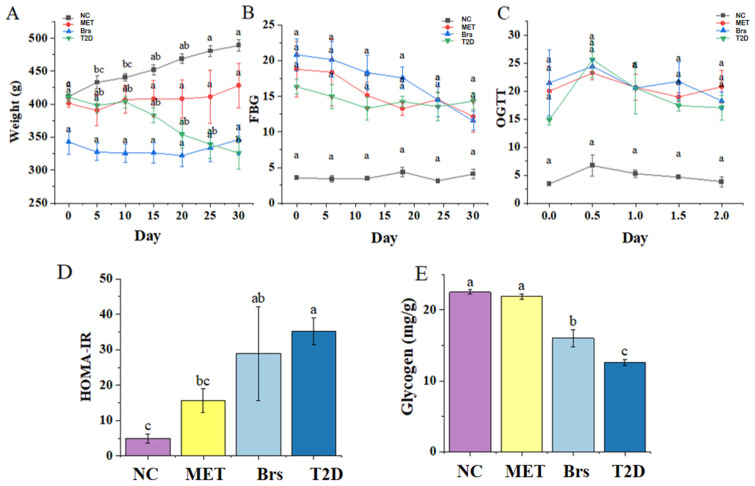
The influence of Brp on body weight (**A**), FBG (**B**), OGTT (**C**), IR (**D**), and glycogen (**E**). Data are expressed as the mean ± SD (n = 3); different superscript letters indicate statistically significant differences among groups (*p* < 0.05). Identical letters denote no significant difference.

Diabetic symptoms are characterized by weight loss accompanied by hyperphagia and polydipsia [28]. Changes in body weight and insulin parameters were used to visually evaluate the effects of Brp and metformin [29]. Figure 1A demonstrates that during the experiment, body weight differed significantly between the NC group and T2D group. Notably, Brp and metformin gavage mitigated diabetes-induced weight loss. FBG and OGTT serve as standard diagnostic tools for diabetes mellitus, effectively identifying individuals with impaired glucose regulation or dysfunctional insulin response [30]. In the NC group, FBG levels remained within the range of 3.4 mmol/L to 4.1 mmol/L (Figure 1B). In contrast, the T2D group’s FBG reached 16.37 mmol/L and never dropped below 13.3 mmol/L. The curves for the MET and Brs groups showed that both Brp and metformin reduced FBG in T2D rats. After 30 days of intervention, FBG levels fell from 18.8 mmol/L to 12.13 mmol/L in the MET, and from 20.83 mmol/L to 11.57 mmol/L in the Brs group. Blood glucose was monitored at 30 min intervals (0–120 min) after oral gavage of 2.0 g/kg glucose (Figure 1C). The NC, MET, and Brs groups showed a similar glycemic pattern: peak blood glucose at 30 min post-dosing, followed by a gradual return to baseline levels, whereas the T2D group exhibited sustained hyperglycemia [31]. These results show that Brp has the ability to relieve the symptoms of impaired glucose tolerance caused by diabetes.

Insulin resistance (IR) describes a state where target organs display reduced sensitivity to insulin, causing a decline in insulin’s capacity to promote the absorption and utilization of glucose in peripheral tissues [32]. Particularly, insulin resistance manifests in liver tissue, featuring compromised glucose utilization and diminished glycogen synthesis [33]. As shown in Figure 1D, the T2D group displayed markedly increased HOMA-IR, almost nine times that of the NC group. This finding indicates that insulin sensitivity in diabetic rats without pharmacological intervention continues to decline. In the Brs group, the HOMA-IR index decreased to 28.87, which was a 17.80% decrease compared to the T2D group. The decline in insulin sensitivity causes an imbalance in glycogen synthesis [34]. From Figure 1E, the glycogen content decreased by 44.23% in the T2D group compared with the NC group. After intervention with Brp, the glycogen content increased by 27.61% compared with the T2D group. These results indicated that Brp not only alleviated the decline in insulin sensitivity but also enhanced glycogen synthesis. The raw data of insulin and hepatic glycogen are presented in Table 1.

### 3.2. Impacts of Brp on Serum Lipids with T2D

**Figure 2:**
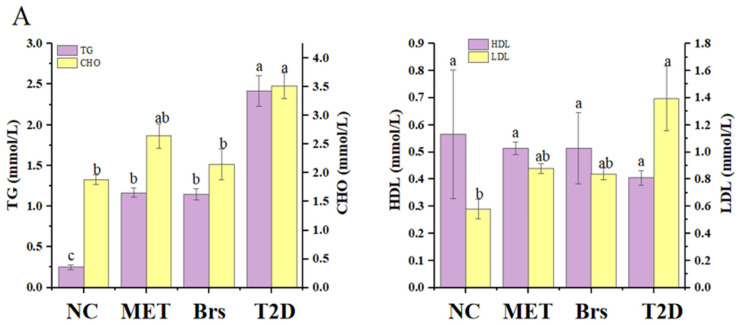

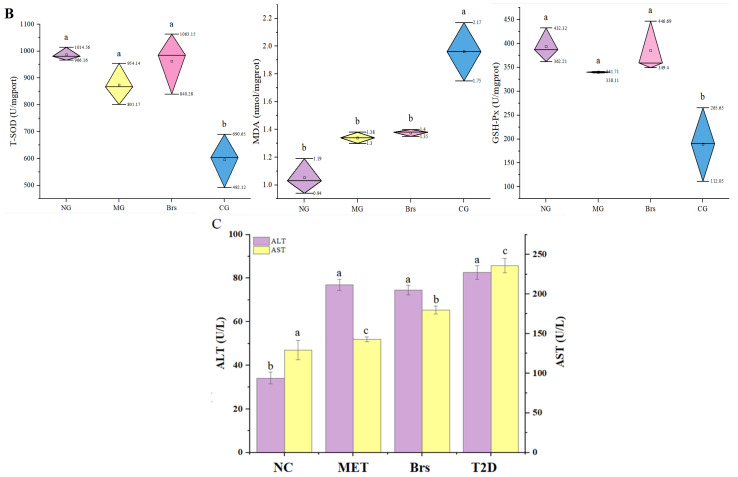
The influence of Brp on the four blood lipid parameters: capacity (**A**), liver antioxidant (**B**), and two key enzymes (**C**). Data are expressed as the mean ± SD (n = 3); different superscript letters indicate statistically significant differences among groups (*p* < 0.05). Identical letters denote no significant difference.

T2D, a chronic metabolic disorder, is frequently associated with dyslipidemia [35]. Prior research has shown that IR (insulin resistance) can induce adipocyte damage, leading to dyslipidemia, which further exacerbates IR itself. Dyslipidemia is typically marked by elevated levels of CHO, TG, LDL-C, and low levels of HDL-C [31]. From Figure 2A, CHO levels and TG levels in the T2D group increased by 86.70% and 864%, while the level of HDL-C decreased by 28.35%, compared with the NC group, indicating lipid metabolism dysfunction in T2D rats. Following a 30-day intervention period, the levels of TG, LDL-C, CHO decreased, and HDL-C levels exhibited an increasing trend. This suggests that Brp possesses the capacity to reduce the LDL-C level and facilitate the transport of TG and cholesterol CHO from the plasma to the liver [36].

### 3.3. Impacts of Brp on Liver-Related Parameters with T2D

The liver serves as one of the primary target organs for insulin action and oxidative stress in diabetes [37]. Restoration of hepatic organ function represents an effective strategy for diabetes treatment [32]. In this work, the activities of T-SOD and GSH-Px (key antioxidant enzymes), MDA (a key product of lipid peroxidation), and ALT and AST (core indicators of liver function) were measured in the livers of rats (Figure 2B,C).

Diabetes mellitus is frequently accompanied by dyslipidemia, which induces substantial free radical generation, impairs antioxidative capacity, and promotes excessive peroxidation product accumulation [38]. After Brp intervention, the activities of GSH-Px and T-SOD were notably elevated (Figure 2B). The activity of GSH-Px in the T2D group decreased to around 51.94% of that in the NC group, and the activity of T-SOD in the T2D group decreased to 39.68% of the NC group, indicating that diabetes will cause damage to GSH-Px and T-SOD activities. In the Brs group, GSH-Px activity increased to 103.54% of the T2D group. T-SOD activity showed a similar trend, increasing significantly by 61.69% compared with the T2D group. As a marker reflecting lipid peroxidation, MDA content was significantly increased in the T2D group, which was 86.13% higher than that in the NC group [23]. In the Brs and MET groups, MDA levels were significantly reduced by 29.59% and 31.63%, respectively. These findings indicate that Brp notably enhanced antioxidant parameters in T2D rats, improving their antioxidant capacity by elevating GSH-Px and T-SOD activities and decreasing MDA levels. These conclusions were consistent with the report from Mondal et al., which indicated that oral administration of *Arctium lappa* L. [39]. Polysaccharides significantly enhanced T-SOD and GSH-Px activities in the livers of immunomodulatory mice, and notably reduced MDA content.

ALT and AST are widely recognized as indicators of hepatocellular injury and liver function [36,40]. As shown in Figure 2C, ALT and AST levels in the T2D group increased by 141.69% and 87.01%, respectively, compared with the NC group. In the MET group, ALT and AST levels decreased by 6.81% and 39.45%, respectively, compared with the T2D group. Notably, Brp exhibited greater efficacy in reducing ALT levels than metformin, with an ALT reduction of 9.80% compared to the T2D group (vs. a 6.81% reduction in the MET group). The raw data of ALT and AST are presented in Table 1.

### 3.4. Microscopic Observation of Tissue Structures

**Figure 3:**
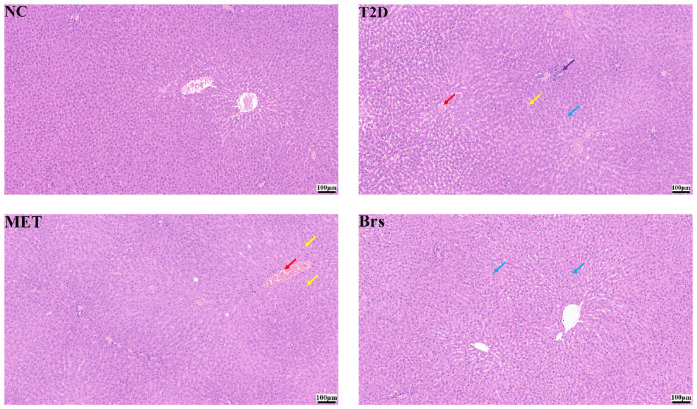
Liver pathological sections. Note: Red arrow: hepatic sinusoidal congestion; blue arrow: hepatocellular edema; yellow arrow: hepatocellular steatosis; purple arrow: inflammatory cell infiltration.

The liver is critical for maintaining blood glucose homeostasis, so observation of liver tissue structure is necessary [41,42]. Liver tissue sections were analyzed by a third-party testing company using a double-blind method; all observations were qualitative (no histopathological scoring system was applied for steatosis, inflammation, or ballooning). As pictured in Figure 3, in the NC group, hepatocytes were rounded and plump; the portal area showed no obvious abnormalities; and no inflammatory cell infiltration was detected. In contrast, in the T2D group, venous and small-area hepatic sinusoidal congestion were observed; occasional hepatocyte edema (swollen cells with loose, lightly stained cytoplasm) was noted; rare steatosis (sparse small circular cytoplasmic vacuoles) was present; and focal lymphocyte-dominated inflammatory cell infiltration was seen in the portal area. Both the Brs and MET groups showed histological improvement. In the Brs group, occasional hepatocyte edema (swollen cells with loose, lightly stained cytoplasm) was observed; the MET group additionally showed venous congestion, rare steatotic hepatocytes, and tiny vacuoles. These findings suggest that Brp alleviated liver tissue lesions associated with T2D. The reduced abundance of *Desulfovibrio* in the Brs group may be one of the microbiota changes associated with improved hepatic status.

### 3.5. Brp’s Impacts on the Composition of T2D Rats’ Gut Microbiota Composition

**Figure 4:**
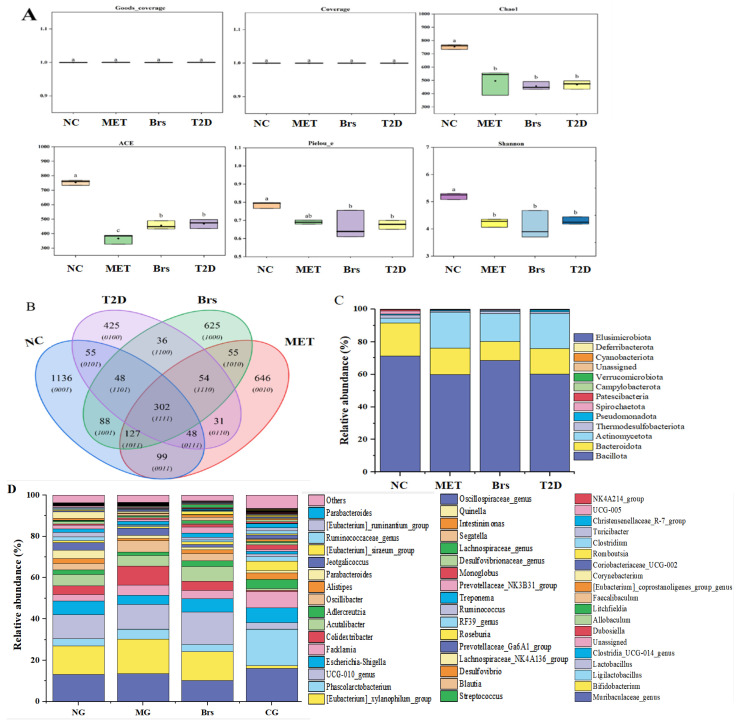
The influence of Brp on intestinal biodiversity in T2D rats; α-diversity (**A**), Venn diagram (**B**); phylum-level stacked bar chart (**C**); genus-level stacked bar chart (**D**). Data are expressed as the mean ± SD (n = 3); different superscript letters indicate statistically significant differences among groups (*p* < 0.05). Identical letters denote no significant difference.

More and more studies reveal that the gut microbiota serves as the cornerstone for addressing numerous diseases [43,44]. Multiple studies show that it is pivotal in the digestion and absorption of nutrients: it first gradually digests polysaccharides into single sugars or oligosaccharide fragments, and then ferments these fragments into SCFAs [45]. High-throughput sequencing was utilized to examine how Brp fermentation impacts the gut microbiota. As shown in Figure 4A, Coverage and Goods’ coverage indices for each group were both close to 1, indicating that the sequencing depth encompassed all species present in the samples [46]. Alpha diversity indices of the various samples were computed under the same sequencing depth. As depicted in Figure 4A, the NC group exhibited the highest Chao, ACE, Peilou_e, and Shannon diversity indices, whereas the MET group showed the lowest values. These phenomena may be induced by the side effects of diabetic illnesses, proving that the T2D models were successfully constructed from a different perspective. With the development of intervention, the values of these indices increased, owing to the Brp and metformin. A comparable phenomenon was observed in polysaccharides derived from *Patinopecten yessoensis* skirt and tea [31,47]. To visualize variations in microbial community composition among different groups, a Venn diagram was constructed for comparative analysis. As presented in Figure 4B, the NC, T2D, Brs, and MET groups contained 1903, 999, 1335, and 1362 operational taxonomic units (OTUs), respectively. These results suggest that Brp and metformin have the ability to restore microbiota diversity to promote the balance of gut. This may contribute to the therapeutic effect of Brp on T2D rats.

To more clearly find out the key microbiota, we conducted a multi-level taxonomic analysis to characterize microbial architecture across groups. As illustrated in Figure 4C, at the phylum taxonomic level, the intestinal flora of all the groups was dominated by *Bacillota*, *Bacteroidetes*, *Actinobacteria*, and *Thermodesulfobacteriota*, which accounted for almost 96% of the total microbial composition, the same as the previous studies [48]. As in previous studies, relative to the NC group, the T2D group demonstrated reduced abundances of *Bacillota* and *Bacteroidetes*, coupled with a pronounced elevation in *Actinomycetota* levels [49]. The percentage of the three microbiota in the Brs group trended towards approaching those in the NC group. The proportions of *Bacillota*, *Bacteroidetes*, and *Actinomycetota* were 68.37%, 11.78%, and 17.04% in the Brs group, whereas they were 60.15%, 15.58%, and 21.57% in the T2D group, respectively. This suggests that Brp has the ability to restore gut homeostasis by decreasing the abundance of *Actinomycetota*. Moreover, in the T2D group, beneficial genera such as *Bifidobacterium*, *Lactobacillus*, *Blautia*, *Roseburia*, *Phascolarctobacterium*, and [*Eubacterium*]_*coprostanoligenes*_group decreased, while harmful microbiota, including *Escherichia-Shigella*, *Desulfovibrio*, and *Streptococcus,* increased (Figure 4D). A prominent characteristic of the gut microbiota in T2D models is the reduced abundance of butyrate-producing bacteria, most notably *Roseburia intestinalis* and *Faecalibacterium prausnitzii* [44,50]. With Brp treatment, the abundances of *Bifidobacterium*, *Lactobacillus*, *Blautia*, *Roseburia*, and other beneficial microbiota increased, and the harmful microbiota decreased. Gram-negative bacterial lipopolysaccharide (LPS) results in the development or worsening of T2D by enhancing intestinal permeability and altering mucosal immune responses, especially when intestinal barrier function is impaired or there is excessive proliferation of Gram-negative bacteria [51,52]. So, the decrease in Desulfovibrio can avoid the deterioration of T2D. Previous studies have shown that *Blautia* successfully inhibited lipid deposition, weight gain and increased blood glucose levels in T2D model mice, and simultaneously regulated the composition of the intestinal microbiota in the mice [53]. With that said, Brp may promote SCFA production by enriching SCFA-producing genera such as *Blautia* and *Roseburia*, while also potentially reducing the risk of metabolic deterioration by decreasing LPS-producing bacteria.

### 3.6. Impacts of Brp on Fecal Metabolic Profiles

**Figure 5:**
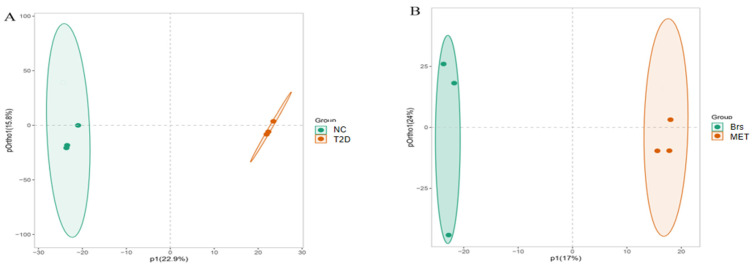

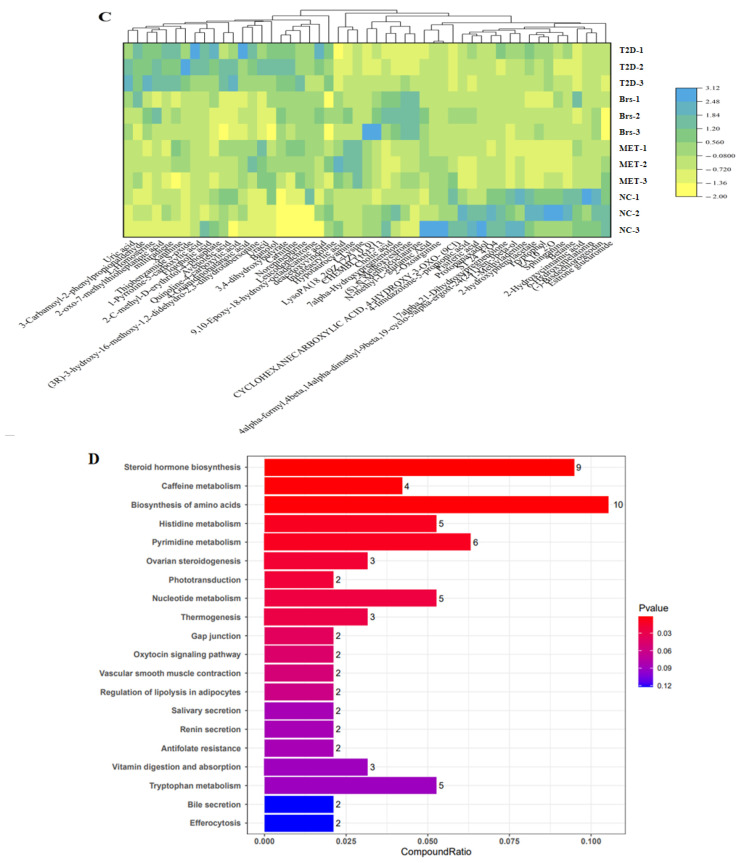

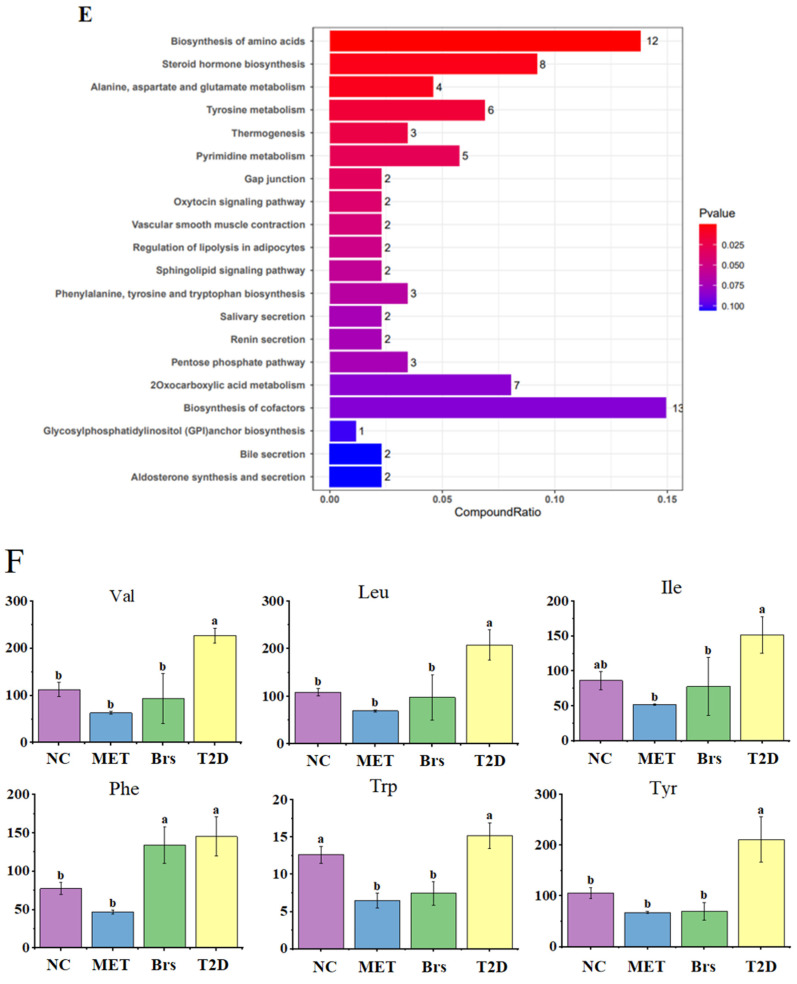
Untargeted metabolomic profiling: OPLS-DA comparison between the NC and T2D groups (**A**). OPLS-DA analysis between Brs and MET (**B**). Heatmap of different metabolites in feces among NC, T2D, Brs, and MET groups (**C**). Bar charts illustrating metabolic pathway influencing factors in NC vs. T2D (**D**) and Brs vs. MET (**E**) comparisons. Amino acid metabolism (**F**). Data are expressed as the mean ± SD (n = 3), different superscript letters indicate statistically significant differences among groups (*p* < 0.05). Identical letters denote no significant difference.

Metabolomics has emerged as a promising approach in precision diabetes care, facilitating the identification of biomarkers for diagnosing the disease, predicting its progression, and guiding management strategies [54]. This is achieved through tailored phenotyping and the continuous tracking of individual responses to therapeutic interventions. While polysaccharides resist digestion and breakdown within the digestive system, they undergo fermentation by gut microorganisms. Metabolites generated through this process potentially exert positive regulatory effects on body weight management, inflammatory reactions, insulin sensitivity, and glucose metabolic balance. OPLS-DA was initially chosen to assess sample and group separability, and the resulting score scatter plots exhibited a clear distinction between the NC and T2D, and Brs/MET groups (Figure 5A,B). Model validation was performed via permutation testing. In the NC/T2D cohort, the intercepts for R^2^Y and Q^2^ were 0.99 and 0.26, respectively, while corresponding values in the Brs/MET cohort were 0.98 and 0.38. Notably, all permuted R^2^Y and Q^2^ values (left distribution) fell below the original data points (right distribution), confirming the absence of overfitting in the OPLS-DA model [31]. Variable importance in projection (VIP) scores were employed to screen for metabolites linked to the initiation, advancement, and remission of diabetes. There were 50 significantly different metabolites exhibited by the heat map.

Figure 5C showed that uric acid, 3,4-dihydroxybutyrate, xanthine, carnitine were substantially heightened in the T2D group, which aligns with the results of prior research investigations [55]. Conversely, treatment groups exhibited an increased concentration of lysophosphatidic acid (18:2(9Z,12Z)/0:0) and indoleacrylic acid, whereas these metabolites were reduced in the T2D group. This phenomenon might be attributed to their roles as precursors of 1-linoleoyl-glycerophosphocholine and indolepropionate, respectively. Notably, indolepropionate can significantly reduce the risk of developing diabetes. Lysophospholipids participate in lipid signaling pathways through interactions with their corresponding receptors, thereby promoting glucose-dependent insulin secretion [56]. In contrast, indolepropionate exerts its preventive effect by inducing intestinal enteroendocrine L cells to release glucagon-like peptide 1. This peptide subsequently facilitates insulin release and enhances insulin sensitivity [57]. Additionally, betaine levels—linked to reduced T2D risk—were notably decreased in the T2D group while increased in the Brs group. That is to say, Brp could reduce the T2D risk by increasing lysophosphatidic acid (18_2(9Z,12Z)_0_0), indoleacrylic acid, and betaine levels and decreasing high-risk metabolites such as 3,4-dihydroxybutyrate, xanthine, and carnitine. Another hypoglycemic mechanism of natural polysaccharides involves the promotion of bile acid synthesis [58]. Cholic acid is an important part of bile acids and also plays a key role in reducing FBG. In our study, the NC group and Brs group had higher cholic acid levels than the T2D group, indicating that Brp could regulate the bile acid cycle. These findings demonstrate that Brp had many different mechanisms in improving T2D symptoms.

Leveraging metabolites with significant alterations, we pinpointed 9 metabolic pathways (*p* < 0.05) markedly impacted by T2D, with only 1 of the top three being amino acid metabolism (Figure 5E). When comparing the NC, T2D, and Brs groups, we found that two of the top three pathways were amino acid metabolism (Figure 5F). Especially, the alanine, aspartate, and glutamate metabolism pathway changed most significantly. Interestingly, new metabolic pathways emerged, like alanine, aspartate and glutamate metabolism, phenylalanine, tyrosine and tryptophan biosynthesis metabolism, tyrosine metabolism, pentose phosphate pathway metabolism and aldosterone synthesis, regulation of lipolysis in adipocytes, and secretion metabolism. These results suggest that Brp could not only influence amino acid metabolism but also affect glycolipid and antioxidant-related metabolism.

Amino acid homeostasis imbalance is also associated with insulin resistance. Elevated dietary intake of branched-chain amino acids (Val, Leu, Ile) might accelerate the onset of insulin resistance (IR) [59], while increased circulatory BCAAs concentrations have also been linked to IR pathogenesis. That may be related to the elevated BCAAs catabolism and the buildup of lipid intermediates, which impairs insulin signaling [60]. In our study, the concentrations of BCAAs, phenylalanine, tryptophan, and tyrosine were markedly elevated in the T2D group compared to the NC group (Figure 5F). With the intervention of Brp, these concentrations decreased and approached those in the NC group. Based on the insulin FBG indices mentioned above, it is hypothesized that branched-chain amino acids (BCAAs) undergo more frequent cycling and decomposition in the T2D group. Another suggestion is that Brp polysaccharides may be involved in regulating gut bacterial populations—including *Lactobacillus* (the most prevalent genus engaged in amino acid catabolism) and *Bacteroides* (which enhances the metabolic efficiency of branched-chain amino acid breakdown)—that are potentially linked to BCAA and AAA metabolism [61]. This is a hypothesis derived solely from correlations between fecal amino acid profiles (not plasma amino acids) and microbiota composition. Notably, the mechanistic links between these fecal amino acid changes and insulin resistance (typically discussed for circulating BCAAs) remain speculative, and the complex bidirectional causality among gut microbiota, amino acid profiles, and host metabolism has not been fully resolved in this model.

### 3.7. Influence of Brp on Gut Microbiota and Its Relationship with Key Metabolites

**Figure 6:**
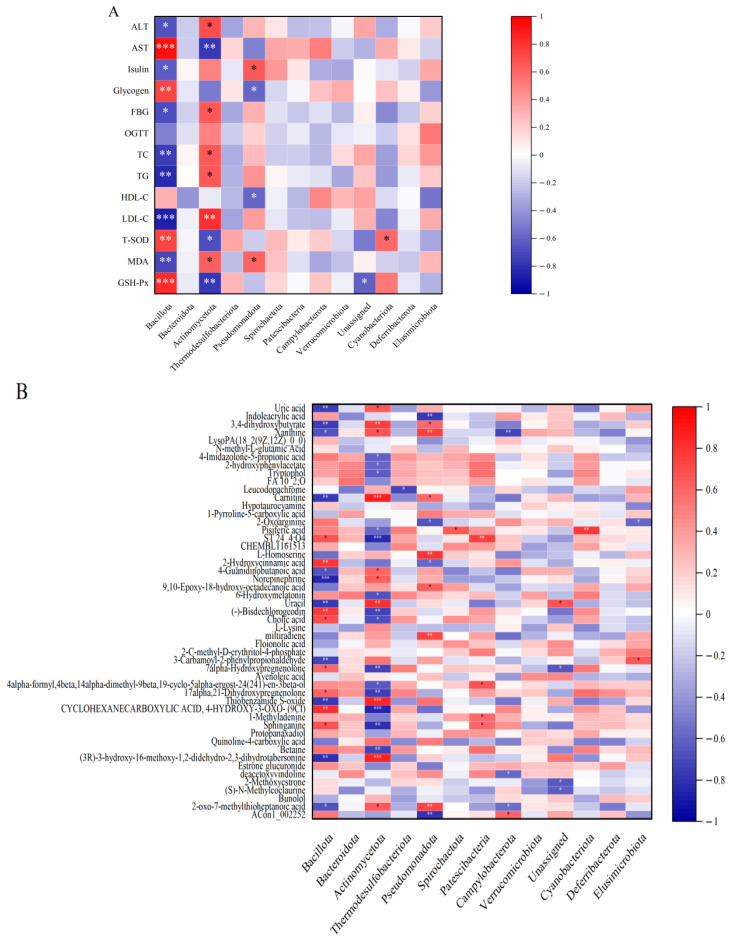
Heatmaps of spearman correlations: gut microbiota vs. biochemical parameters (**A**) and gut microbiota vs. metabolites (**B**). Symbols indicate differences relative to the control group: * *p* < 0.05, ** *p* < 0.01, *** *p* < 0.001.

Numerous studies link obesity, type 2 diabetes (T2D), and other diseases to gut microbiota balance, with gut microbiota imbalance exacerbating hyperglycemia, insulin resistance, dyslipidemia, and hepatorenal dysfunction. To assess Brp’s direct regulatory effect on gut microbiota, we analyzed correlations between phylum-level microbial abundance and these indicators. As shown in Figure 6A, *Bacillota*, *Actinomycetota*, *Pseudomonadota*, and *Cyanobacterota* were tightly associated with the above-mentioned indicators. Notably, *Bacillota*, *Actinomycetota*, and *Pseudomonadota* exerted distinct effects: *Bacillota* was significantly positively correlated with glycogen, AST, T-SOD, and GSH-Px, and significantly negatively correlated with FBG, insulin, MDA, LDL-C, TC, TG, and ALT. This is inconsistent with the findings of Zang et al. [62], regarding the effects of red quinoa polysaccharide on type 2 diabetic mice. Specifically, red quinoa polysaccharide inhibited the progression of diabetes by reducing the abundances of norank_f_*Muribaculaceae* and *Lachnospiraceae*_NK4A136_group, while increasing the relative abundances of *Akkermansia*, unclassified_f_*Lachnospiraceae*, norank_f_*Eubacterium_*coprostanoligenes_group, *unclassified_f_Atopobiaceae*, and norank_f_*Lachnospiraceae*. This discrepancy may be attributed to differences in the structural characteristics of polysaccharides derived from distinct sources. This suggested that *Bacillota* may reduce FBG by regulating glycogen metabolism and increasing the activity of antioxidant enzymes (T-SOD and GSH-Px), while the positive correlation with AST may reflect improved liver function. In contrast, *Actinomycetota* was significantly positively correlated with MDA, LDL-C, TG, TC, and FBG, indicating a potential role in exacerbating metabolic dysfunction. *Pseudomonadota* generally had the same effect as *Actinomycetota*, whereas *Cyanobacterota* had the same effect as *Bacillota*.

To further explore associations between altered gut microbiota and microbial metabolites, we correlated phylum-level microbial abundance with levels of the top 50 significantly differential metabolites (Figure 6B). It had the same trend as microbial abundance and above indicators: *Bacillota* was significantly negatively related to uric acid, 3,4-dihydroxybutyrate, xanthine, carnitine, norepinephrine, uracil, and 3-carbamoyl-2-phenylpropionaldehyde, but significantly positively related to ST 24_4;O4, 2-hydroxycinnamic acid, (-)-bisdechlorogeodin, cholic acid, 7alpha-Hydroxypregnenolone. *Bacillota* and *Actionmycetota* exhibited opposite trends. Other microbiota were also vital to research, like *Pseudomonadota*. It was significantly positively related to 3,4-dihydroxybutyrate, xanthine, carnitine, L-homoserine, miltiradiene, 2-oxo-7-methylthioheptanoic acid, but negatively related to indoleacrylic acid, 2-oxoarginine, 2-hydroxycinnamic acid, and ACon1_002252.

## 4. Conclusions

This study investigated Brp’s ameliorative effects on HFD/STZ-induced T2D. The results showed that Brp exerted multiple therapeutic effects in T2D rats: lowering FBG, stabilizing weight, enhancing OGTT, alleviating insulin resistance, promoting hepatic glycogen synthesis, upregulating hepatic antioxidant enzyme activities, and mitigating oxidative stress-induced damage. Brp regulated gut microbiota composition by reducing harmful taxa (e.g., *Actinomycetota*, *Desulfovibrio*) and increasing beneficial bacteria (e.g., *Roseburia*, *Bifidobacterium*, *Lactobacillus*). Additionally, Brp reduced T2D risk by increasing protective metabolites (lysophosphatidic acid 18:2, indoleacrylic acid, betaine, cholic acid) and decreasing high-risk metabolites (3,4-dihydroxybutyrate, xanthine, carnitine). Abnormal metabolism of BCAAs is closely correlated with energy balance, obesity, and metabolic disorders. In our study, Brp alleviated the imbalance of BCAA metabolism in T2D by reducing the levels of valine, leucine, isoleucine, phenylalanine, tyrosine, and tryptophan. This research provides preclinical evidence that Brp may ameliorate T2D-related metabolic disturbances in rats and supports further investigation of Brp as a candidate ingredient for diabetes-oriented functional foods. Future investigations will focus on elucidating its structure–activity relationships and underlying antidiabetic mechanisms.

In this study, all outcome assessments (including histology scoring and oral glucose tolerance test/OGTT curve analysis) were performed by investigators blinded to group allocation. Specifically, a third party assigned random codes to samples prior to analysis, and the codes were only unblinded after data collection was completed to ensure the implementation of blinding. Furthermore, we acknowledge that the small sample size (n = 3 per group) may limit the generalizability of our findings and increase the risk of unstable effect estimates. However, we confirm that the data in this study have undergone multiple testing corrections, particularly for gut biodiversity and untargeted metabolomics analyses, and our interpretation focused on consistent and biologically meaningful results. We regret that we did not measure these parameters in the current study due to logistical constraints during sample collection and analysis (limited sample volume for multi-omics profiling, unavailability of validated assay kits for GLP-1 at the time of experimentation). Notably, in rodent studies, Brp has shown promise as a functional supplement for type 2 diabetes (T2D). However, its therapeutic potential in humans requires rigorous clinical and translational research to validate its efficacy and safety, suggesting that Brp may be a potential functional ingredient with T2D therapeutic potential—though the specific dose–response relationship warrants further investigation. Finally, since this experiment is still ongoing and involves subsequent studies by other team members, the data cannot be publicly disclosed at this time. Nevertheless, access to the data is available to readers upon reasonable request.

## Figures and Tables

**Table 1:** Raw data of ALT, AST, insulin, and hepatic glycogen in T2D models treated with Brp.

Group	ALT (U/L)	AST (U/L)	Insulin (mU/L)	Hepatic Glycogen (mg/g)
NC-1	37.609	246.01	26.97	22.82
NC-2	28.97	217.45	28.30	22.23
NC-3	35.959	243.93	26.61	22.56
MET-1	77.27	147.98	27.20	21.56
MET-2	81.35	143.03	34.86	22.32
MET-3	72.33	137.320	27.06	21.90
Brs-1	71.914	183.52	42.68	16.21
Brs-2	78.977	169.95	46.93	17.14
Brs-3	72.648	185.71	36.34	14.78
T2D-1	79.293	110.903	52.42	12.09
T2D-2	88.812	152.224	59.52	12.61
T2D-3	79.71	124.793	54.42	13.01

## Data Availability

The original contributions presented in the study are included in the article, further inquiries can be directed to the corresponding author.
